# Supplementary material for: Testing the accuracy of 3D automatic landmarking via genome-wide association studies
Source: G3 (Bethesda). 2021 Dec 31;12(2):jkab443. doi: 10.1093/g3journal/jkab443 (PMC9210295; doi:10.1093/g3journal/jkab443)
Supplement: jkab443_Supplemental_Material_Legends [file jkab443_supplemental_material_legends.docx]

**Supplementary material**

**Table S1**. Definition of landmarks used in this study (same as in Pallares et al. 2015).

**Table S2**. Two-way mixed model ANOVA and Procrustes ANOVA for digitizing error.

**Table S3.** Univariate and multivariate mapping - SNP locations. QTLs identified in this study and overlapping ones found in this present re-analysis of data from Pallares et al. (2015). CS: Centroid size, chr: chromosome, pos: position in bp, log10p: log10-transformed association P values for each mapping, Lower.CI and Upper.CI: Bayesian estimates of their confidence intervals.

**Supplementary data.** Data generated via the automatic phenotyping method subsequently used in both the univariate and multivariate QTL mapping (file ‘pheno.auto.skull.mand.csv’). This file contains principal component (PC) scores and centroid size (CS) for skull and lower jaws.
